# Histopathologic Effects of *Dirofilaria immitis* Microfilaria on Internal Organs of Dog Confirming by PCR Technique

**Published:** 2012

**Authors:** AO Ceribasi, S Simsek

**Affiliations:** 1 Department of Pathology, Faculty of Veterinary Medicine University of Firat, 23119, Elazig-TURKEY; 2 Department of Parasitology, Faculty of Veterinary Medicine University of Firat, 23119, Elazig-TURKEY

**Keywords:** *Dirofilaria immitis*, PCR, Histopathology, Paraffin, Dog

## Abstract

**Background:**

The heartworm disease is an infectious disease of dogs with *Dirofilaria immitis* combined with cardiovascular and circulatory abnormalities. The heartworm disease can become a serious health risk when associated with a severe infection. In this study, a male, 8 year-old dog that died suddenly was necropsied and all tissues were examined grossly.

**Methods:**

Major organs including heart, lungs, liver, spleen, kidneys, brain, eyes, and testis were fixed in 10% neutral formalin, embedded in paraffin, sectioned at 5-µm thickness, stained with hematoxylin and eosin, and examined with a light microscope. For each examined organ, paraffin-embedded tissues were cut and placed in eppendorf tubes for genomic DNA extraction. PCR was performed using two sets of primers for amplification of a 302 bp ITS-2 gene fragment and a 203 bp cytochrome oxidase subunit 1 (*CO1*) gene fragment of *D. immitis*.

**Results:**

During the necropsy examination, 46 adult *D. immitis* were found in the portal vein, right ventricle, and atrium of the heart and pulmonary trunk. Microscopically, microfilarias were found throughout the vessels of different organs including lungs, kidneys, liver, heart, brain, and spleen. All tissues examined by PCR were positive for *D. immitis* ITS-2 and *CO1*.

**Conclusion:**

PCR technique now represents an effective method for identification of *D. immitis* from formalin-fixed samples.

## Introduction

Dirofilariasis, caused by *Dirofilaria immitis*, exists worldwide, but the endemic areas are those with high temperatures and mosquito vector populations. Adult worms live in the pulmonary arteries; females produce microfilariae, which are taken up by mosquitoes, which transmit the infection to other susceptible animals. In dogs, the infection is chronic and leads to congestive heart failure. *Dirofilaria immitis* infection is characterized by several different clinical pictures, caused by both adults and microfilariae (1). *D. immitis* in dogs can be diagnosed through careful morphological examination of circulating microfilariae, detection of circulating antigens, histochemical or immuno-histochemical staining of circulating microfilariae or, more recently, through molecular approaches. Morphological identification of circulating microfilariae requires special fixation protocols and expertise and is less accurate than genotyping (2, 3). The usefulness of PCR methods for the identification of *D. immitis* microfilaria in dog blood (the definitive host) has been reported in several recent publications (2–4), however there are no reports of genotyping from paraffin-embedded tissues.

The aim of this case report was to establish a PCR protocol for the detection and identification of *D. immitis* from dog with formalin-fixed, paraffin-embedded tissues as a source of DNA. We examined a necropsied dog with histologically confirmed dirofilariasis to compare the accuracy of PCR based tools.

## Materials and Methods

An intact male, 8 years-old Labrador (*Canis lupus familiaris*) was referred to the Pathology Department of Firat University for postmortem examination from a farm in Bingöl Province where in the eastern Turkey. The dog born in coastal region of Bursa Province, western Turkey and one year ago transferred to the Bingol. According to personal information, the animal had been inappetant, coughing, with acute severe respiratory distress and hematuria. The dog died suddenly and was submitted for necropsy. Systemic necropsy was performed and all tissues were examined grossly and major organs including heart, lungs, liver, spleen, kidneys, brain, eyes and testis were fixed in 10% neutral formalin, embedded in paraffin, sectioned at 5 µm thickness and stained with hematoxylin and eosin and examined with a light microscope.

For each organ, 10 µm-thick sections from paraffin-embedded tissues were cut and placed in 1.5 ml eppendorf tubes. To avoid cross-contamination of samples, the microtome blade was cleaned with xylene between each block. Sections were deparaffinized with 1 ml xylene for 10 min at 37 °C. Subsequently, samples were centrifuged at 4000 rpm for 5 min and the supernatant was removed. This procedure was repeated once. After deparaffinization, rehydration in 100%, 90%, 80% and 70% ethanol followed. Thereafter 70% ethanol was removed and tissue lysis solution was added (Fermentas DNA Purification Kit). For DNA extraction, we chose a commercially available DNA extraction kit based on a simple salting out procedure (5). We used the manufacturers’ protocol for paraffin-embedded tissues with a modification of the proteinase K step, namely, an overnight digestion and addition of 25 µl aliquots of proteinase K (20 mg/ml). The incubation was performed with constant agitation at 56 °C. After extraction, the DNA pellet was air-dried and resuspended in 50 µl sterile distilled water and stored -20 °C. DNA was also extracted from the adult worm as mentioned above for use as a positive control.

The PCR was performed using two sets of primers. Initially, we used one primer pair: 5'-CATCAGGTGATGATGTGATGAT-3' (forward) and 5'-TTGATTGGATTTTAACG TATCATTT-3' (reverse), which have been described by Mar et al., (6) for the amplification of a 302 bp ITS-2 gene segment. A second set of primers was used: 5'-AGTGTAGAGGGTCAGCCTGAGTTA-3' (forward) and 5'-ACAGGCACTGACAATA CCAAT-3' (reverse), identical to those described in a previous study (3) for the amplification of a 203 bp cytochrome oxidase subunit 1 (*CO1*) gene fragment of *D. immitis*. Positive control DNA was used for each PCR and distilled water was used as a negative control. The amplified products were separated by gel electrophoresis in 2.5% agarose gel with a Tris-boric acid-EDTA (TBE, pH 8.3) buffer at 90 V for 30 min. Following electrophoresis, the amplified products were visualized with ethidium bromide staining for 30 min at room temperature.

Subsequently, 203 bp bands were cut from the gel and amplified DNA fragments were purified by QIAquick Gel Extraction Kit (Qiagen, USA). The CO1 sequences were automatically obtained using a 377 ABI PRISM system (Applied Biosystems). Nucleotide sequence analysis was undertaken by BLAST algorithms and databases from the National Center for Biotechnology (http://www.ncbi.nlm.nih.gov).

## Results

During the necropsy examination, 46 adults *D. immitis* were found in right ventricle and atrium of the heart and portal vein lumen. The right ventricle and atrium were enlarged. Pulmonary emboli, containing dead parasites, were seen in a small number of pulmonary arterial branches, and some areas of the lungs were congested.

**Fig. 1:**
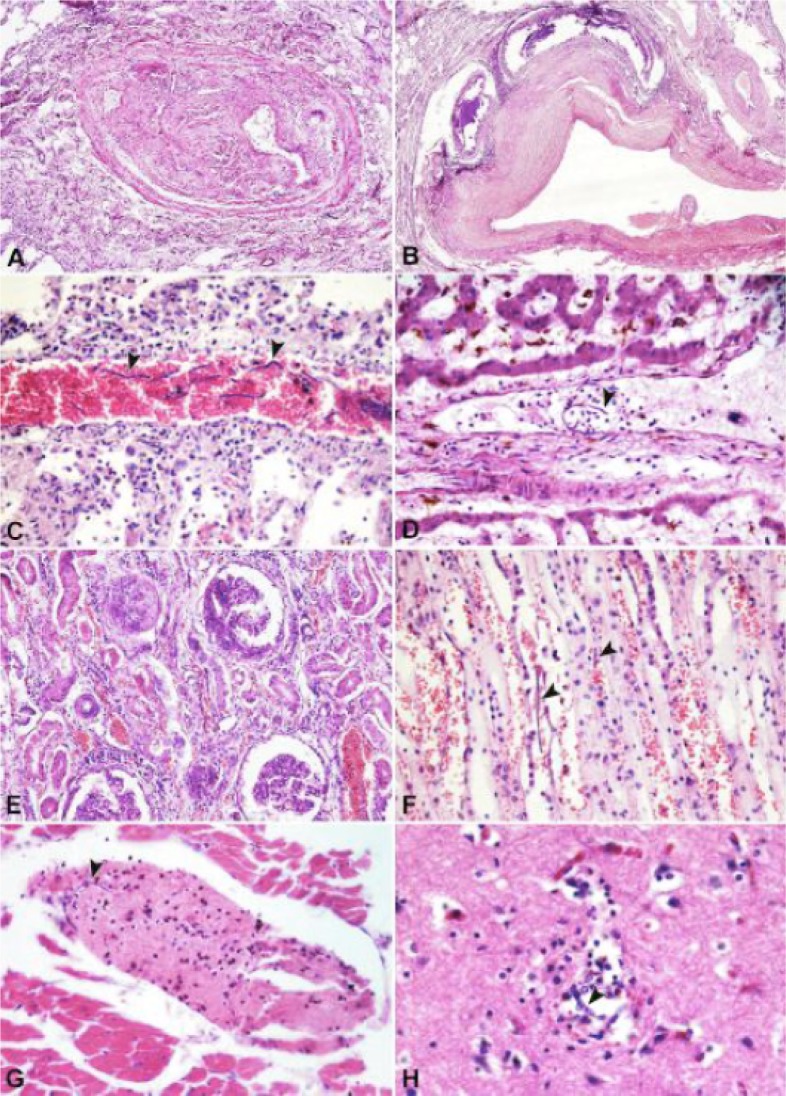
A. Partial vaso-oclusive changes in pulmonary artery, H&E X20, B. Villous endarteritis and granulomatous reactions large pulmonary artery (arrows), H&E X20. Microfilaria presentation in pulmonary (C), liver (D), kidney (F), heart (G), brain (H) vessels (arrows) H&E (C, D, F, G X100; H X200); E: Chronic membranoproliferative glomerulonephritis and interstitial nephritis, H&E X50

Microscopically, microfilarias were found throughout the vessels of different organs including lungs, liver, kidneys, heart, brain, and spleen (Fig. 1, C, D, F, G, H). The most significant lesion observed in the lungs was vascular lesions. These lesions included obstructive fibrosis of peripheral pulmonary arteries and villous endarteritis (Fig. 1, A, B). In the kidneys, marked chronic membranoproliferative glomerulonephritis as well as chronic interstitial nephritis, with multiple cortical and medullary scarring was found (Fig. 1, E). Additionally, microfilariae were observed within cerebral capillaries and surrounded by microglial cells.

**Fig. 2:**
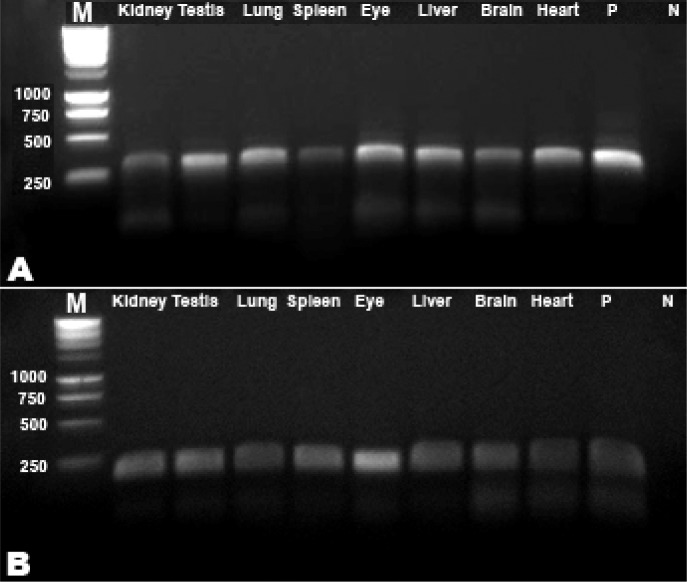
Species specific PCR amplification of *Dirofilaria immitis* A: ITS-2 (302 bp) and B: CO1 (203 bp). M: Molecular weight marker, P: Positive control, N: Negative control

All examined tissues were found positive by ITS and CO1-PCR. A 302 bp band consistent with the ITS-2 gene product was observed in all patient samples (Fig. 2A), while a 203 bp band was observed for CO1-PCR (Fig. 2B). Examined CO1 sequence (GenBank accession no: JF304903) was identified as corresponding to the *D. immitis* with 100% identity with previously published sequences.

## Discussion

Heartworm infection in dogs has been diagnosed around the globe. Relocation of infected, microfilaremic dogs appears to be the most important factor contributing to further spreading of the parasite. The prevalence and spread of heartworm infection in Turkey has been comprehensively studied by both microfilarial detection and serological-molecular methods (4, 7). An increasing number of cases are now being diagnosed in Turkey and Europe (8, 9). Thus, accurate clinical and histopathological diagnosis of *D. immitis* is important for development of an effective treatment programme.

In canine dirofilariasis, the lungs are variably but consistently affected with changes consisting of dilatation, hypertrophy, thrombosis, myointimal proliferation of the pulmonary artery, infarction, hemorrhage, hemosiderosis, chronic inflammation, and fibrosis (1). We additionally observed granulomatous reactions in perivascular connective tissue and bronchial epithelial changes, findings that are consistent with the presence of dead intrapulmonary *D. immitis* worms. On the other hand, changes were also observed in large pulmonary arteries, which were not infected with parasites. *Wolbachia* is an intracellular, Gram-negative bacteria belonging to the order Rickettsiales. Many pathogenic filarial worms of humans and animals harbor *Wolbachia*, and evidence strongly suggests that there is an endosymbiotic relationship between the bacteria and its filarial host (10). Recent interest has also been directed toward the role *Wolbachia* might play in the inflammatory pathology characteristic of filarial infection. It is widely accepted that *Wolbachia* is released into the tissues of the filarial-infected host following worm death and that bacteria-derived molecules provoke innate inflammatory responses (11). It was considered that pulmonary changes might have been produced by *Wolbachia* in pulmonary artery.

Investigators have sought to develop a reliable and practical diagnostic test for identification of filariae on histological sections (12). Although these investigators succeeded in performing PCR on fresh tissues or those fixed in ethyl alcohol, they were unable to reproduce the test on formalin-fixed tissues. This is a serious drawback, for biopsies are normally sent to the histologist already fixed in 10% formalin. Because the diagnosis of dirofilariasis might not be suspected antemortem, fixation of excised nodules in methyl alcohol or submission of unfixed samples is uncommon. Our PCR technique now represents an effective method for identification of *D. immitis* from formalin-fixed samples. The PCR technique here described show that this method yields positive results from various formalin-fixed tissues.

The PCR method used in our study might be of value in the analysis of infected clinical or pathological samples from various organs, other than the lungs. The determination of the sensitivity and specificity of this method requires additional large-scale testing of tissues from known infected and non-infected dogs.
